# Exploring stress, cognitive, and affective mechanisms of the relationship between interpersonal trauma and opioid misuse

**DOI:** 10.1371/journal.pone.0233185

**Published:** 2020-05-15

**Authors:** Jessica Roberts Williams, Veronica Cole, Susan Girdler, Martha Grace Cromeens

**Affiliations:** 1 School of Nursing, University of North Carolina at Chapel Hill, Chapel Hill, North Carolina, United States of America; 2 Department of Psychology, Wake Forest University, Wake Forest, North Carolina, United States of America; 3 Department of Psychiatry, University of North Carolina at Chapel Hill, Chapel Hill, North Carolina, United States of America; Yale University, UNITED STATES

## Abstract

**Background:**

People with a history of interpersonal trauma, including intimate partner violence, sexual assault, and adverse childhood experiences, are disproportionately affected by the current opioid epidemic. Interpersonal trauma has been shown to increase risk for chronic pain conditions, prescription opioid use, and opioid misuse. Stress, cognition, and affective function have been examined as potential mechanisms that may influence opioid misuse among individuals with a history of interpersonal trauma. However, no studies have examined these factors simultaneously, despite their interrelatedness.

**Objective:**

The purpose of this study was to 1) examine perceived stress, perceived cognitive function, depressive symptoms, and PTSD symptoms as potential mechanisms of opioid misuse among individuals with a history of interpersonal trauma, 2) examine the types of interpersonal trauma that are associated with opioid misuse, and 3) assess the mediating role of pain and opioid prescription.

**Methods:**

A cross-sectional, observational study design was conducted. Data were collected through a confidential self-report online survey using validated instruments (n = 230). A series of regression analyses were conducted to identify mechanistic factors and interpersonal trauma types associated with opioid misuse, opioid prescription, and pain intensity. Structural equation modeling was used to examine mediating effects of pain intensity and opioid prescription.

**Results:**

Opioid prescription, depressive symptoms, and intimate partner violence increased the odds of reporting opioid misuse. Pain intensity and adverse childhood experiences increased the odds of opioid prescription. Higher levels of perceived stress and depressive symptoms were associated with increased pain intensity. Pain intensity emerged as a mediator of the relationship between depressive symptoms and opioid misuse.

**Conclusions:**

Our work shows that there are likely several pathways through which interpersonal trauma can lead to opioid misuse. Interventions aimed at improving depressive symptoms and coping with traumatizing events should be included as part of comprehensive trauma-informed pain management practices.

## Introduction

The misuse of opioids has reached epidemic proportions with an estimated 130 people dying every day in the United States due to opioid overdose [1]. The rapid increase in medical opioid prescriptions for the treatment of pain in the 1990s and 2000s has been identified as a major driver of this public health emergency [2]. People with a history of interpersonal trauma (IPT), including intimate partner violence, sexual assault, and adverse childhood experiences, are disproportionately affected by the current opioid epidemic [3, 4]. IPT is extremely prevalent in society (up to 60% of adults will report at least one IPT experience [5, 6]) and has been shown to increase risk for pain conditions [7–9], prescription opioid use [3, 10, 11], and opioid misuse [4, 12, 13]. Specifically, persons who experience IPT are 3.35 times more likely to have a chronic pain condition [7], twice as likely to be prescribed opioids [3], and have a 4.5 greater odds of developing an opioid use disorder [4] than those without a history of IPT. Despite the established pathway between IPT, pain, opioid prescription, and opioid misuse, we still know little about underlying mechanisms that contribute to this risk. A better understanding of these factors is critical for the development of effective interventions aimed at preventing opioid misuse, particularly among vulnerable populations.

Mental health conditions, such as depression and post-traumatic stress disorder (PTSD), are a common consequence of IPT [14–17] and one of the most well established risk factors for opioid misuse [18, 19]. Studies have shown that individuals with a history of IPT, who are currently prescribed opioids or are opioid dependent, have higher rates of mental health conditions [20, 21]. Balousek and colleagues [20] found that among primary care patients currently prescribed opioids, those reporting higher psychiatric symptoms (i.e., depression, anxiety, hallucinations,concentration/memory, violent behavior, and suicide) were 5 times more likely to report any abuse in the past 30 days and twice as likely to report lifetime abuse for each half point increase on the Addiction Severity Index Psychiatric Scale. Schäfer et al. [21] showed that among individuals receiving outpatient treatment for opioid dependence, victims of sexual violence were more likely to suffer from severe to extreme psychological distress (females: 66.3% vs. 46.7%, OR = 2.5, 95%-CI = 1.7–3.3; males: 60.7% vs. 40.8%, OR = 2.5, 95%-CI = 1.7–3.3).

Given this increased risk, most literature examining mechanisms of the relationship between IPT and opioid misuse has focused on the mediating role of mental health conditions, with mixed results [13, 22–24]. Two studies showed that mood and anxiety disorders partially mediated the relationship between adverse childhood experiences and opioid misuse [13, 22], whereas another study found that PTSD did not mediate the relationship between interpersonal violence and opioid misuse behaviors [24]. These discrepancies may be due to differences in the type of mental health condition or IPT examined. These findings also indicate that there are likely other mechanisms that may account for the relationship between IPT and opioid misuse. Austin and Shanahan [23] used data from the *National Longitudinal Study of Adolescent to Adult Health* to examine depressive symptoms and pain as mediators of the relationship between adverse childhood experiences and opioid misuse. They found that pain, but not depressive symptoms, in adolescence was a significant mediator. Other studies have shown that depression and PTSD symptoms mediate the relationship between IPT and chronic pain outcomes, demonstrating the importance of accounting for both mental health and pain when examining the effects of IPT on opioid misuse [25–29].

Stress may also play a role in the relationship between IPT and opioid misuse. Exposure to chronic stress, such as IPT, can lead to dysregulation in stress-responsive biological systems, resulting in increased immune/inflammatory activity from chronic, stress-induced activation of the HPA axis [30–35]. This chronic, systemic state of inflammation alters central pain processing mechanisms through the overactivation of spinal cord nociceptors and has been implicated in numerous chronic pain conditions [36–38]. Stress may also influence opioid misuse behaviors. Preclinical and clinical studies show that the endogenous opioid system plays an important role in regulating physiological responses to stress and opioid medications may help individuals cope with stressors by inhibiting stress-related symptoms [39–41]. Stress has also been shown to influence cravings in individuals receiving treatment for opioid dependence, leading to subsequent misuse [42, 43]. Garami and colleagues [44] specifically examined the impact of perceived stress on the relationship between IPT and opioid misuse. They found that individuals receiving treatment for opioid dependence reported greater incidence and severity of IPT and higher perceived stress levels compared to controls. Severity of IPT, however, was a stronger predictor of dependence status than perceived stress.

Impairments in cognitive functioning may also serve as a mechanism through which IPT contributes to opioid misuse. Strong links have been established between IPT and diminished cognitive functioning (e.g., intellectual performance, executive functioning, reward processing) [15, 45]. A substantial body of evidence also demonstrates the impact substance use can have on subsequent cognitive functioning [46, 47]. The nature of cognitive deficits seen with chronic substance use are dependent on the type of drug, environment, and genes of the user, but often result in impairments in cognitive flexibility, working memory, and attention [46]. In addition, research demonstrates that there may also be cognitive vulnerabilities that increase the risk for initiation of drug use and the development of substance use disorders [47, 48, 49]. These include general cognitive dysfunction and those associated with specific personality traits, including sensation-seeking, impulsivity, and behavioral disinhibition [47, 48, 49]. The relationship between cognitive function and opioids, specifically, is less clear. In an updated systematic review of 25 studies, Højsted and colleagues [50] found evidence of opioid induced cognitive deficits among patients with cancer pain, but not among patients with non-cancer pain. Other studies have also shown little to no relationship between opioids and cognitive functioning and, when a relationship is found, it is often attributed to mental health or pain sequalae [51, 52, 53]. Additional research is needed to further clarify potential relationships between cognitive functioning and opioid use, particularly among vulnerable populations.

In addition to the potential roles that mental health, stress, and cognitive function may play in the relationship between IPT and opioid misuse, prior research has shown that the type of IPT experienced can influence opioid misuse risk [4, 12]. These studies indicate that cumulative trauma (i.e., experiencing multiple types of IPT or higher numbers of IPT incidents) and exposure to IPT earlier in life increases risk for opioid misuse. Another study examined the effects of cumulative trauma on the likelihood of being prescribed opioids [10]. This study found that among women with a history of intimate partner violence, experiences of adverse childhood experiences increased risk for opioid prescription.

Despite the extant evidence, no studies have comprehensively examined potential mechanisms that may influence opioid misuse among individuals with a history of IPT. As previously noted, stress, cognitive function, and mental health are interrelated, and each has differential impacts on pain, prescription opioid use, and opioid misuse. Thus, it is important to examine these factors simultaneously to understand better the direct and indirect effects each has on opioid use behaviors. This study extends existing knowledge by testing a comprehensive model, the purpose of which is to identify potential mechanisms of the relationship between IPT and opioid misuse. Specifically, the following research questions were addressed:

1. Are perceived stress, perceived cognitive function, depressive symptoms, and PTSD symptoms associated with opioid misuse after adjusting for pain intensity, opioid prescription, type of IPT, and demographic variables?
2. Which specific forms of IPT (i.e., intimate partner violence, sexual assault, and adverse childhood experiences) are associated with opioid misuse, opioid prescription, and pain intensity after adjusting for mechanistic factors (i.e., perceived stress, perceived cognitive function, depressive symptoms, and PTSD symptoms) and demographic variables?
3. Does pain intensity or opioid prescription mediate the relationship between mechanistic factors (i.e., perceived stress, perceived cognitive function, depressive symptoms, and PTSD symptoms) and opioid misuse after adjusting for type of IPT and demographic variables?

## Methods

### Study design

A cross-sectional, observational study design was used to meet the aims of this study. Data were collected through a confidential self-report online survey completed at one timepoint. The study protocol was approved by the University of North Carolina at Chapel Hill Institutional Review Board before engaging in study activities (18–1507). Electronic informed consent was obtained from all participants prior to data collection activities.

A convenience sample was recruited from the central North Carolina region using community-based and online recruitment strategies. Study advertisements were posted in general community locations (e.g., coffee shops, libraries) and distributed through research volunteer listservs. Recruitment materials were also posted in places likely to be frequented by our target population, including pain management clinics, substance abuse treatment centers, and domestic violence shelters. Recruitment materials described the research as examining how certain types of trauma can impact health and stated that an individual must have experienced domestic violence, sexual assault, or abuse as a child in order to participate. Recruitment materials avoided mentioning opioids so those without a history of opioid use would still participate.

### Sample and setting

To be eligible to participate in the study, participants had to be ≥18 years old, able to complete a survey in English, and report a history of at least one type of IPT (i.e., intimate partner violence, sexual assault, and/or adverse childhood experiences). Potential participants were screened for eligibility through a brief questionnaire prior to engaging in the study survey. Age and ability to complete a survey in English were assessed through two questions. Interpersonal trauma was assessed using six items from the Stressful Life Events Screening Questionnaire (SLESQ) [54]. These items measure six types of interpersonal traumas (sexual assault (penetration); attempted sexual assault; molestation; child physical assault; adult physical assault by an intimate partner; threatened with weapon by an intimate partner). The SLESQ has adequate psychometric properties (convergent validity: r = .77, k = .64; test-retest reliability: r = .89, k = .73) [54].

### Data collection procedures

Data were collected from July 2018 to September 2018 through a self-report survey administered through the Qualtrics® online survey platform. Recruitment materials provided instructions for accessing the survey through a personal computer, tablet, or phone as well as information for contacting study staff if they needed assistance with access. The first screen provided participants with general information about the study purpose (i.e., to learn more about how certain types of trauma can impact health). This screen also stated that the study involves asking personal questions about current and past experiences with different types of IPT and asked participants to ensure they were in a safe and private location before moving forward with the survey. Individuals who were still interested in participating moved to the next screen to complete the eligibility questionnaire. Eligible individuals then advanced to the informed consent screen (those who did not meet eligibility criteria did not move on to the survey). After reviewing the informed consent information, those who were still interested in participating and provided consent proceeded to the study survey. After completing the survey, participants received a $25 gift card by email or postal mail as compensation for their time. Several strategies were taken to prevent individuals from completing the survey multiple times or changing their eligibility criteria in order to access the survey. These included restricting the survey so it could only be completed once from a particular IP address, including a CAPTCHA question, and monitoring for duplicate or similar email/mailing addresses.

### Measures

The following measures were selected to assess constructs of interest in this study. Instruments were evaluated for psychometric quality upon selection and reevaluated in this study.

#### Demographic characteristics

Participants self-reported demographic information including age, sex, and race/ethnicity.

#### Intimate partner violence

The *Revised Conflict Tactics Scale—Victimization* (CTS2; 32 items, α = .96) was used to measure the lifetime occurrence of violence victimization within intimate relationships [55, 56]. The CTS2 is a well-established instrument with demonstrated convergent, discriminant, and factorial validity [55, 56]. It assesses violence across four domains: psychological aggression, physical assault, sexual coercion, and injury. Participants were asked to report the number of times they experienced each item (never, 1 time, 2 times, 3 or more times). A total sum score was calculated across items with scores ranging from 0 to 96.

#### Sexual assault

The *Sexual Experiences Survey*, *Short Form Victimization* (SES-SFV, 35 items, α = .96) was used to identify unwanted sexual experiences since the age of 14, including unwanted sexual contact, attempted coercion, coercion, attempted rape, and rape [57]. The SES-SF is established with demonstrated test-retest reliability and convergent validity among adult women and to a lesser extent among men [57–60]. Participants were asked to report the number of times they experienced each item (never, 1 time, 2 times, 3 or more times). A total sum score was calculated across items with scores ranging from 0 to 105.

#### Adverse childhood experiences

The *Adverse Childhood Experiences Scale* (ACE, 17 items, α = .87) examines childhood exposure to abuse, including experiences of psychological, physical, and sexual abuse, violence against the mother, and living with household members who were substance abusers, mentally ill or suicidal, or imprisoned [61]. The ACEs scale is established with demonstrated criterion validity [61, 62] and test-retest reliability [63] in community and clinical samples of adults. Participants were asked to indicate if they experienced each item (yes/no). A total sum score was calculated across items with scores ranging from 0 to 17.

#### Perceived stress

The *NIH Toolbox Perceived Stress Scale* (10 items, α = .90) assesses the frequency of perceived stress in the past month [64]. This scale has established concurrent, discriminant, and factorial validity in clinical and non-clincial samples [65, 66]. Questions focus on the participant’s feelings and level of control in situations of acute change or mounting problems. Responses are based on a 5-point Likert style scale ranging from “Never” to “Very Often”. A total sum score was calculated with scores ranging from 10 to 50.

#### Cognitive functioning

The *PROMIS® Cognitive Function 8a Scale* (8 items, α = .96) was used to assess perceived cognitive deficits and the extent to which cognitive impairments interfere with daily functioning [67]. The scale has demonstrated adequate reliability, construct validity, and factorial validity in clinical samples [68–70]. Responses are based on a 5-point Likert style scale ranging from “Never” to “Always”. A total sum score was calculated with scores ranging from 8 to 40.

#### Depressive symptoms

The *PROMIS® Depression 8a Scale* (8 items, α = .96) was used to assess symptoms of emotional distress and depression occurring in the past 7 days [71, 72]. The scale demonstrated adequate reliability and construct validity in clinical and non-clinical samples [72–74]. Responses are based on a 5-point Likert style scale ranging from “Never” to “Always”. A total sum score was calculated with scores ranging from 8 to 40.

#### PTSD symptoms

The *PTSD Checklist for DSM-5* (PCL-5, 20 items, α = .96) was used to assess the occurrence of 20 symptoms over the past month, which correspond to PTSD diagnostic criteria in the DSM-5 [75, 76]. The PCL-5 demonstrated strong internal consistency, test-retest reliability, and convergent and discriminant validity among trauma exposed college students, veterans, and military service personnel [75, 77, 78]. Responses are based on a 5-point Likert style scale ranging from “Not at All” to “Extremely”. A total sum score was calculated with scores ranging from 0 to 80. The National Center for PTSD suggests a cut-off score of 31–33 on the PCL-5 as indicative of probable PTSD diagnosis. In this study, we were interested in how symptom severity, rather than PTSD diagnosis, impacts relationships in the model. Thus, we examined this scale as a continuous measure of PTSD symptoms instead of applying diagnostic criteria. PTSD symptoms were not linked to a Criterion A stressor as detailed in the DSM-5, thus could be in response to the IPT experiences reported during eligibility screening or another stressor.

#### Pain intensity

The *PROMIS® Pain Intensity* measure (1 item) was used to assess pain intensity. [79] This measure asks participants to identify their pain intensity in the last seven days with a visual analog scale ranging from no pain (0) to worst pain (10).

#### Opioid prescription and opioid misuse

Opioid prescription was assessed by asking participants if they had received a prescription for pain medication from a medical provider in the past year (yes/no). Those who responded yes were asked to report on their prescription opioid use behaviors using the *PROMIS® Prescription Pain Medication Misuse 7a Scale* (7 items, α = .94) [80, 81]. Convergent, content, concurrent, and factorial validity has been established for this scale in clinical and non-clinical samples [80, 82]. This scale asks participants to report the frequency of common prescription medication misuses in the past year. Illicit opioid use was assessed by asking participants to report on past year heroin use (yes/no) and past year use of a prescription pain medication that was not prescribed to them by a health provider (yes/no). Opioid misuse was classified as any indication of illicit opioid use or obtaining a score one standard deviation or higher than the mean (≥ 21) on the *PROMIS® Prescription Pain Medication Misuse 7a Scale*. This classification of opioid misuse (i.e., the misuse of prescription pain relievers or the use of heroin) is based on the definition of opioid misuse used by the Substance Abuse and Mental Health Services Administration in the *2018 National Survey on Drug Use and Health* [83].

### Statistical analyses

Descriptive statistics were calculated and bivariate correlations conducted to examine the relationships between variables. A series of regression analyses were conducted to address our first two research questions; specifically, to identify mechanistic factors (i.e., perceived stress, perceived cognitive function, depressive symptoms, and PTSD symptoms) and IPT types (i.e., intimate partner violence, sexual assault, and adverse childhood experiences) associated with 1) opioid misuse; 2) opioid prescription; and 3) pain intensity. Each model adjusted for demographics (i.e., age, sex, and race/ethnicity). All variables were added simultaneously to each regression model and chosen based on the results of our bivariate correlation analyses and because all have been established as a potential mechanism or confounder in prior research.

**Fig 1:**
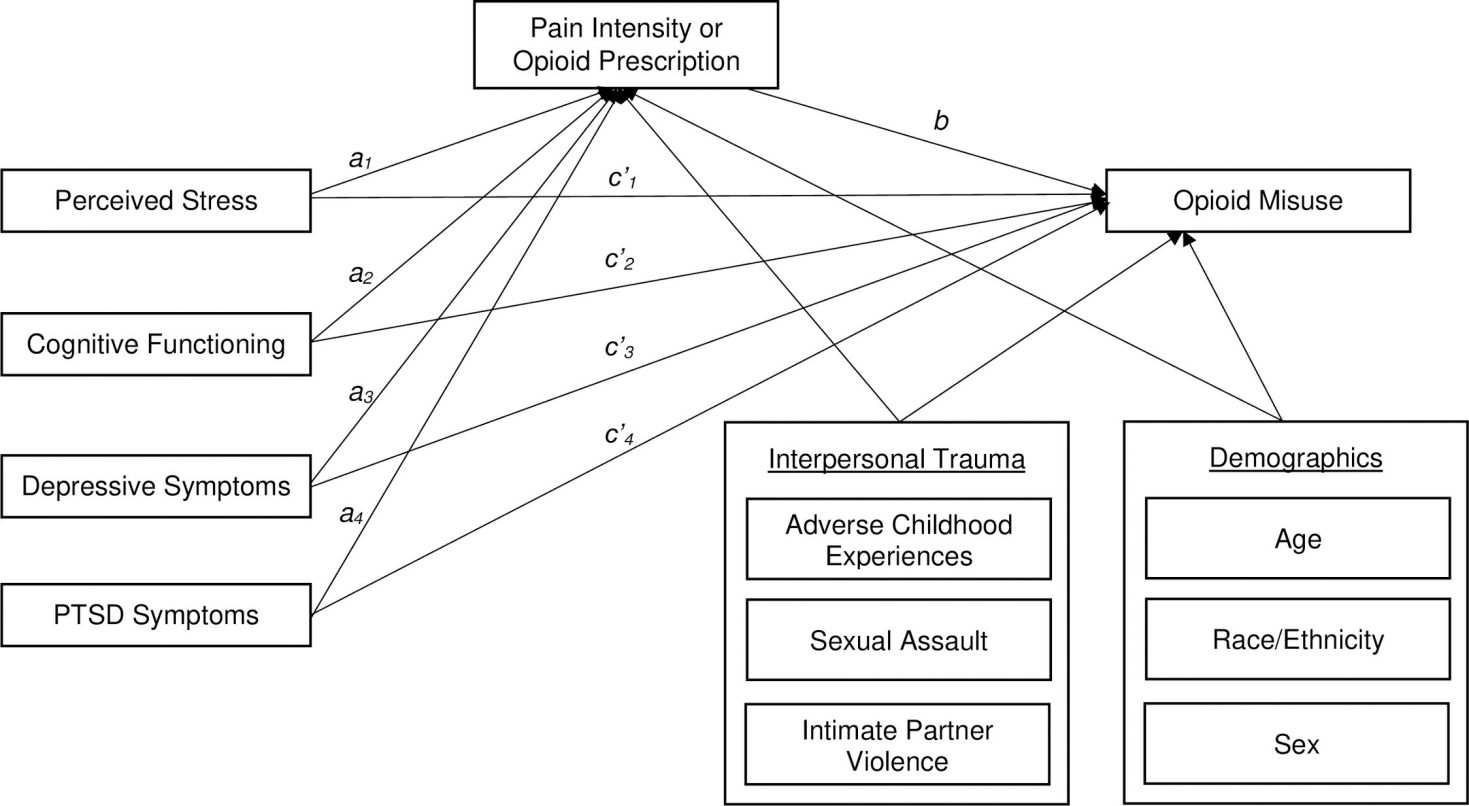
Fitted structural equation models for testing mediation.

After conducting regression analyses, we addressed Question 3 –whether pain intensity or opioid prescription mediate the relationship between mechanistic factors and opioid misuse, controlling for IPT and demographic variables–using the structural equation models shown in Fig 1. Two models were run, one with opioid prescription as the mediator and the other with pain intensity as the mediator. This was done because we did not want to hypothesize any relationships between opioid prescription and pain intensity, which are very highly correlated. Because two endogenous variables were binary (opioid prescription and opioid misuse), a logistic link function was used to model these variables using the diagonally weighted least squares (WLSMV) estimator. [84] Importantly, these models do not contain any paths between variables that cannot be inferred from the individual regression models described above. However, the mediation model was run for the purpose of obtaining accurate standard errors for the indirect effect, which is calculated as the product of the effect of the predictor on the mediator (e.g., perceived stress on pain intensity) and the mediator on the outcome (e.g., pain intensity on opioid misuse). Standard errors, which are necessary for significance testing, may be inaccurate when the indirect effect is calculated from two separate regression models [85]. Thus, the model in Fig 1 was fit and confidence intervals were obtained using the bias-corrected bootstrap method [86]. As is standard in mediation models, which contain all possible effects from predictors to the mediator and outcome, the models we tested here are saturated and no estimates of fit statistics are generated. In addition, standardized estimates are reported instead of odds ratios in order to maintain comparability to paths predicting continuous outcomes. Descriptive statistics and regressions were conducted using R [87], and the structural equation models were fit using Mplus [84].

## Results

### Descriptive statistics and correlations

Participant characteristics are presented in Table 1. Two-hundred fifty-two individuals participated in the survey. Twenty-two participants were excluded due to missing data, resulting in a final sample size of 230. The average age of participants was 28.59 (SD = 10.90), 61.7% were female, 57.4% were White, Non-Hispanic, 16.1% African American/Black, 11.7% Asian, 8.7% Hispanic/Latino, and 6.8% identified other racial/ethnic origins. About 35% reported that they had received an opioid prescription from a medical provider in the past year and almost one quarter (23.9%) reported some type of opioid misuse behavior.

**Table 1:** Participant characteristics (n = 230).

	Mean (SD)	N (%)
Age	28.59 (10.90)	
Sex		
Male		79 (34.3)
Female		142 (61.7)
Transgender/Other		9 (3.9)
Race/Ethnicity		
Asian		27 (11.7)
African American/Black		37 (16.1)
White, Non-Hispanic		132 (57.4)
Hispanic/Latino, White		20 (8.7)
Multiple Races/Other		14 (6.1)
Interpersonal Trauma		
Intimate Partner Violence	27.79 (23.92)	
Sexual Assault	22.23 (26.15)	
Adverse Childhood Experiences	6.33 (4.46)	
Perceived Stress	30.56 (8.14)	
Perceived Cognitive Function	27.25 (8.79)	
Depressive Symptoms	19.02 (9.27)	
PTSD Symptoms	26.41 (18.99)	
Pain Intensity	3.41 (2.82)	
Opioid Prescription		
No		149 (64.8)
Yes		81 (35.2)
Opioid Misuse		
No		175 (76.1)
Yes		55 (23.9)

Table 2 presents the bivariate correlations between study variables. Significant correlations were seen between opioid misuse, opioid prescription, and pain intensity. Mechanistic factors (i.e., perceived stress, perceived cognitive function, depressive symptoms, and PTSD symptoms) were significantly related to each other. Mechanistic factors were also associated with pain intensity and opioid misuse, except for PTSD symptoms, which was not associated with opioid misuse. None of the mechanistic factors were associated with opioid prescription. Intimate partner violence and adverse childhood experiences were associated with all variables of interest. Sexual assault was only associated with intimate partner violence, adverse childhood experiences, PTSD symptoms, and opioid misuse.

**Table 2:** Bivariate correlations between study variables (n = 230).

	1	2	3	4	5	6	7	8	9
1. Intimate Partner Violence									
2. Sexual Assault	.599**								
3. Adverse Childhood Experiences	.268**	.184**							
4. Perceived Stress	.197**	.020	.501**						
5. Perceived Cognitive Function	-.133*	.012	-.400**	-.724**					
6. Depressive Symptoms	.219**	.061	.429**	. 750**	-.549**				
7. PTSD Symptoms	.247**	.186**	.475**	. 682**	-.580**	.707**			
8. Pain Intensity	.275**	.037	.424**	.476**	-.377**	.480**	.309**		
9. Opioid Prescription	.181**	.074	.272**	.094	-.105	.107	.118	.384**	
10. Opioid Misuse	.317**	.143*	.276**	.216**	-.158*	.273**	.103	.349**	.312**

**p ≤ 0.01

* *p* ≤ 0.05

### Relationship between mechanistic factors and IPT types on opioid misuse, opioid prescription, and pain intensity

The results of our regression models are shown in Table 3. Opioid prescription (OR = 4.04, 95% CI = 1.77–9.60, *p* = 0.001), depressive symptoms (OR = 1.10, 95% CI = 1.03–1.19, *p* = 0.009), and intimate partner violence (OR = 1.02, CI = 1.00–1.05, *p* = 0.022) increased the odds of reporting opioid misuse. PTSD symptoms were negatively associated with opioid misuse (OR = 0.95, 95% CI = 0.92–0.99, *p* = 0.006) indicating that as PTSD symptoms decreased the odds of reporting opioid misuse increased. Pain intensity (OR = 1.40, 95% CI = 1.20–1.66, *p* < 0.001) and adverse childhood experiences (OR = 1.10, 95% CI = 1.01–1.20, *p* = 0.036) increased the odds of having an opioid prescription. The relationship between opioid prescription and opioid misuse seen in the previous analysis was also found in this model (OR = 4.42, 95% CI = 1.92–10.54, *p* = 0.001). Higher levels of perceived stress (*β* = 0.09, 95% CI = 0.02–0.15, *p* = 0.009) and depressive symptoms (*β* = 0.08, 95% CI = 0.03–0.13, *p* = 0.002) were associated with increased pain intensity. The relationship between pain intensity and opioid prescription seen in the previous analysis was also found in this model (*β* = 1.37, 95% CI = 0.75–1.99, *p* < 0.001). No IPT variables were associated with pain intensity after adjusting for other factors.

**Table 3:** Regression models for associations between mechanistic factors and opioid misuse (n = 230).

Predictors	Opioid Misuse			Prescription Opioid Use			Pain Intensity		
	OR	CI	p	OR	95% CI	p	β	95% CI	p
Perceived Stress	0.99	0.90–1.10	0.913	0.92	0.85–1.00	0.060	0.09	0.02–0.15	**0.009**
Perceived Cognitive Function	0.99	0.93–1.05	0.733	1.00	0.95–1.06	0.986	-0.01	-0.06–0.03	0.551
Depressive Symptoms	1.10	1.03–1.19	**0.009**	0.95	0.89–1.01	0.094	0.08	0.03–0.13	**0.002**
PTSD Symptoms	0.95	0.92–0.99	**0.006**	1.03	1.00–1.06	0.074	-0.02	-0.04–0.00	0.091
Intimate Partner Violence	1.02	1.00–1.05	**0.022**	1.01	0.99–1.03	0.418	0.00	-0.01–0.02	0.747
Sexual Assault	1.00	0.98–1.02	0.996	0.99	0.97–1.01	0.393	0.01	-0.01–0.02	0.398
Adverse Childhood Experiences	1.10	0.99–1.22	0.079	1.10	1.01–1.20	**0.036**	0.03	-0.05–0.11	0.402
Pain Intensity	1.14	0.96–1.37	0.139	1.40	1.20–1.66	**<0.001**	--	--	--
Opioid Prescription	4.04	1.77–9.60	**0.001**	--	--	--	1.37	0.75–1.99	**<0.001**
Opioid Misuse	--	--	--	4.42	1.92–10.54	**0.001**	0.62	-0.13–1.36	0.105

Adjusted for age, sex, and race

### Pain intensity and opioid prescription as mediators of the relationship between mechanistic factors and opioid misuse

A full listing of the results from both structural equation models is given in the S1 Table and summarized below.

#### Model with pain intensity as a mediator

Standardized estimates of paths from all of the mechanistic variables to pain intensity (labeled *a*_*1*_ through *a*_*4*_ in Fig 1) were calculated. The paths from perceived stress (*a*_*1*_ = 0.214, CI = 0.008–0.420, *p* = 0.042), depressive symptoms (*a*_*3*_ = 0.305, CI = 0.146–0.464, *p* < .001), and PTSD symptoms (*a*_*4*_ = -0.162, CI = -0.315 –-.009, *p* = .038) were significantly different from zero. With respect to the direct effects of each of these mechanistic variables on opioid misuse (labeled *c’*_*1*_ through *c’*_*4*_ in Fig 1), depressive symptoms directly increased the probability of opioid misuse (*c’*_*3*_ = 0.349, CI = 0.016–0.682, *p* = .041) and PTSD symptoms decreased the probability of opioid misuse (*c’*_*4*_ = -0.341, CI = -0.611 –-0.071, *p* = .014). There were no significant effects of perceived cognitive functioning on either pain intensity or opioid misuse. The positive relationship between pain intensity and opioid misuse was significantly different from zero (*b* = 0.243, CI = 0.071–0.415, *p* = 0.006). Note that this is different from the regression analyses, in which this effect did not reach statistical significance, likely owing to the presence of opioid prescription in the regression model and its absence here. Of the indirect effects (here denoted *ab*_1_ –*ab*_*4*_), only the indirect effect of depressive symptoms was significantly different from zero (*ab*_*3*_ = 0.074, CI = 0.005–0.143, *p* = .033).

#### Model with opioid prescription as a mediator

In the model in which opioid prescription was the mediator of the relationship between mechanistic variables and opioid misuse, none of the paths from each of these variables to opioid prescription (labeled *a*_*1*_ through *a*_*4*_ in Fig 1) were significantly different from zero. Direct effects of mechanistic variables on opioid misuse (labeled *c’*_*1*_ through *c’*_*4*_ in Fig 1), mirrored those in the model which included pain intensity as a mediator, with only depressive symptoms directly increasing the probability of opioid misuse (*a*_*3*_ = 0.413, CI = 0.094–0.732, *p* = .011) and PTSD symptoms decreasing the probability of opioid misuse (*a*_*4*_ = -0.394, CI = -0.657 –-0.131, *p* = .003). The positive relationship between opioid misuse and opioid prescription (labeled *b* in Fig 1) was significant (*b =* 0.433, CI = 0.247–0.619, *p* < .001). None of the indirect effects from mechanistic variables (i.e., *ab*_1_ –*ab*_*4*_) were significant.

## Discussion

The purpose of the present study was to identify potential mechanisms of opioid misuse and assess the mediating role of pain and opioid prescription among individuals with a history of IPT. Not surprisingly, our study found a strong link between pain intensity and opioid prescription. Our results also showed that opioid prescription was associated with reported opioid misuse behaviors, providing support for the pathway between pain intensity, opioid prescription, and opioid misuse. Depressive symptoms and, to a lesser extent, perceived stress emerged as possible mechanisms of these relationships. Results also suggest that pathways to opioid misuse among survivors may differ depending on the type of IPT experienced.

### Depression and perceived stress as potential mechanisms of opioid misuse

Depressive symptoms was the only mechanism directly associated with opioid misuse after controlling for other factors and was also indirectly associated with opioid misuse through pain intensity. The associations between depressive symptoms, opioid misuse, and pain intensity among individuals with a history of IPT are consistent with the extant literature [13, 22, 25, 29]. Although we cannot determine directionality of these relationships through our cross-sectional design, prior epidemiologic research provides strong evidence to suggest that depression and substance abuse often occur subsequent to traumatic experiences [14, 88].

Perceived stress was also directly associated with pain intensity in our analyses; however, we did not find evidence that pain mediates the relationship between perceived stress and opioid misuse. Given the cross-sectional nature of this study, we cannot determine if increased stress causes increased pain intensity or if increased stress is a result of higher pain intensity. Both of these potential pathways are supported through prior research [89]. According to the stress model of chronic pain, pain is seen as a type of stressor that increases strain on an individual [89, 90]. This, in turn, increases an individual’s allostatic load and can result in compromised well-being [91]. Other models show that exposure to chronic stress can precede chronic pain and serve as a trigger for pain symptoms [36–38]. It is likely that these conceptualizations of the relationship between stress and pain are not mutually exclusive, but rather, represent a maladaptive cycle. Longitudinal studies are needed to understand better how pain affects subsequent adaptive capacity after exposure to a preceding stressor such as IPT.

It is also important to note that this study used a self-report measure of perceived stress during the past month as an indicator of a person’s adaptive capacity overload [66]. While perceived stress has been shown to be related to chronic pain in prior research [92, 93], other types of measurement, such as biomarkers, can provide additional information regarding the specific biological processes that contribute to the relationship between stress, inflammation, and chronic pain conditions. While the measurement and interpretation of stress and inflammation biomarkers is complex and should be conducted with a clear understanding of their limitations [94–97], future research would benefit from including biomarkers to more specifically examine physiologic changes that can lead to chronic pain.

### PTSD symptoms and perceived cognitive functioning not supported as potential mechanisms of opioid misuse

PTSD symptoms and perceived cognitive functioning were not associated with any of the outcomes examined in the regression models, with the exception of an observed negative relationship between PTSD symptoms and opioid misuse. This negative relationship may be due to the high co-occurrence between PTSD and depressive symptoms or relatively low level of PTSD symptoms in our sample (M = 26.41, SD = 18.99) [98]. McCall-Hosenfeld and colleagues [29] found a similar finding in their study examining PTSD, depression, and substance abuse as potential mediators of the relationship between IPT and somatic symptom severity. Based on a structural equation model, they found that depression, but not PTSD was associated with somatic symptom severity.

The lack of evidence supporting relationships between PTSD symptoms and perceived cognitive functioning with opioid misuse and pain intensity is noteworthy given prior research in this area. Several studies have demonstrated associations between PTSD symptoms and, to a lesser extent, cognitive functioning, with opioid misuse and pain intensity among individuals with a history of IPT. These studies, however, often do not account for co-morbidities, such as depressive symptoms, which may confound observed relationships [26–28]. Given the role of depressive symptoms found in the current study, it is critical that future research account for this to further disentangle the mechanistic pathways between IPT and opioid misuse.

### Unique contributions of IPT types to opioid misuse risk

Previous studies have found that cumulative exposure to IPT and experiencing IPT earlier in life increases risk for opioid prescription and opioid misuse [4, 10, 12]. Our findings extend this work by providing evidence that there may be different pathways to opioid misuse depending on the type of IPT experienced. Intimate partner violence was the only type of IPT directly associated with opioid misuse after controlling for other factors. Prior research shows that individuals in abusive relationships are more likely to be coerced or forced into using substances by their abusive partner which may explain, in part, this finding [99]. This result may also reflect an increased likelihood of individuals to use substances as a form of “self-medication” to cope with emotional distress [100].

Adverse childhood experiences was directly associated with opioid prescription after controlling for other factors. This is consistent with a prior study by Wuest and colleagues [10], which found that among women survivors of intimate partner violence, those with a history of child abuse were more likely to be taking prescription pain medications. Since adverse childhood experiences happen earlier in life, it may be that individuals with these experiences have more time to develop and seek treatment for physical symptoms that would result in opioid prescription. Wuest et al. [10] also found lower rates of over-the-counter pain medication use in their sample, a factor not examined in the current study, but one that may help explain some of the direct association between adverse childhood experiences and opioid prescription. Individuals with a history of childhood trauma often show greater sensitivity to pain and lower pain thresholds [101, 102]. This alteration in pain processing may lead individuals to seek prescription pain relievers rather than over-the-counter options.

### Limitations

The results of this study should be interpreted in light of its limitations. While this study provides evidence of potential mechanisms of the relationship between IPT and opioid misuse, the cross-sectional nature of the study prevents us from making conclusions about the temporal sequence of variables and establishing causality [103]. Thus, the main goal of these analyses were simply to quantify the indirect effects between mechanistic variables and opioid misuse through its proposed mediators. Longitudinal studies are needed to specifically examine the timing of these variables in order to make more definitive statements about cause and effect relationships. In addition, our recruitment and sampling design may have also introduced limitations regarding the generalizability of results. Participants were recruited through convenience sampling methods and, while the demographic characteristics are similar to the population where the study took place, they are likely different from populations in other regions. We also did not collect information on participants’ treatment histories (e.g., currently engaged in treatment for pain management or substance use). Individuals who are currently engaged in clinical or social services may be different with regards to their substance misuse as well as clinical reports around the severity of trauma and depression, compared to those who are not engaged in services. These potential differences should be examined and accounted for in future research. This project was also advertised as a study examining the effects of IPT on health. As such, individuals who expressed interest in participating are likely those who self-identify as having a history of IPT. Given the wide variation in how IPT is defined, some people who have experienced victimization may not recognize it as IPT, and thus, these individuals may be underrepresented in this study. In addition, participants were required to have internet access in order to complete the study survey. While efforts were made to assist individuals with obtaining internet access if needed, some individuals may have been dissuaded from participating due to this barrier.

## Conclusions

Understanding mechanisms that can lead to opioid misuse among IPT survivors is critical for the development of targeted interventions. Our work shows that there are likely several pathways through which IPT can lead to opioid misuse. Depressive symptoms were a salient mechanism of opioid misuse in this study, highlighting the importance of accounting for both physical and affective dimensions when providing treatment for individuals with a history of IPT. Interventions aimed at improving depressive symptoms and coping with traumatizing events should be included as part of comprehensive trauma-informed pain management practices.

## Supporting information

S1 Table Structural equation model results: Pain intensity and prescription opioid use as mediators of the relationship between mechanistic factors and opioid misuse. (DOCX)

S2 Table De-identified data set. (XLSX)
