# Supplementary material for: Exploring stress, cognitive, and affective mechanisms of the relationship between interpersonal trauma and opioid misuse
Source: PLoS One. 2020 May 15;15(5):e0233185. doi: 10.1371/journal.pone.0233185 (PMC7228080; doi:10.1371/journal.pone.0233185)
Supplement: S1 Table — (DOCX) [file pone.0233185.s001.docx]

**S1 Table.** Structural equation model results: pain intensity and opioid prescription as mediators of the relationship between mechanistic factors and opioid misuse

|  |  |  | Model with Opioid Prescription as Mediator | | | | | | | | Model with Pain Intensity as Mediator | | | | | | | |
| --- | --- | --- | --- | --- | --- | --- | --- | --- | --- | --- | --- | --- | --- | --- | --- | --- | --- | --- |
|  |  |  | Paths Predicting Opioid Prescription | | | | Paths Predicting Opioid Misuse | | | | Paths Predicting Pain Intensity | | | | Paths Predicting Opioid Misuse | | | |
|  |  |  | Std. Effect | 95% CI | | P value | Std. Effect | 95% CI | | p value | Std. Effect | 95% CI | | p value | Std. Effect | 95% CI | | p value |
|  |  |  |  | Lower | Upper |  |  | Lower | Upper |  |  | Lower | Upper |  |  | Lower | Upper |  |
| Demographics | | |  |  |  |  |  |  |  |  |  |  |  |  |  |  |  |  |
|  | Age | | 0.153 | -0.010 | 0.316 | 0.067 | -0.121 | -0.323 | 0.081 | 0.243 | 0.272 | 0.170 | 0.374 | <.001 | -0.121 | -0.319 | 0.077 | 0.231 |
|  | Race/Ethnicity | |  |  |  |  |  |  |  |  |  |  |  |  |  |  |  |  |
|  |  | Asian | 0.056 | -0.113 | 0.225 | 0.516 | -0.022 | -0.194 | 0.150 | 0.806 | 0.052 | -0.064 | 0.168 | 0.378 | -0.010 | -0.196 | 0.176 | 0.915 |
|  |  | Black | 0.065 | -0.100 | 0.230 | 0.437 | 0.054 | -0.103 | 0.211 | 0.497 | 0.047 | -0.051 | 0.145 | 0.345 | 0.071 | -0.088 | 0.230 | 0.379 |
|  |  | Hispanic/Latinx | -0.075 | -0.267 | 0.117 | 0.444 | 0.230 | 0.038 | 0.422 | 0.019 | 0.037 | -0.086 | 0.160 | 0.558 | 0.188 | 0.010 | 0.366 | 0.038 |
|  |  | Other Race | 0.061 | -0.102 | 0.224 | 0.463 | -0.150 | -0.366 | 0.066 | 0.172 | 0.096 | 0.000 | 0.192 | 0.051 | -0.147 | -0.365 | 0.071 | 0.185 |
|  | Sex | |  |  |  |  |  |  |  |  |  |  |  |  |  |  |  |  |
|  |  | Female | 0.011 | -0.183 | 0.205 | 0.913 | -0.012 | -0.216 | 0.192 | 0.910 | -0.168 | -0.291 | -0.045 | 0.008 | 0.034 | -0.180 | 0.248 | 0.758 |
|  |  | Other Sex | 0.138 | -0.009 | 0.285 | 0.066 | -0.055 | -0.226 | 0.116 | 0.529 | -0.041 | -0.157 | 0.075 | 0.485 | 0.015 | -0.173 | 0.203 | 0.880 |
| Mechanistic Variables | | |  |  |  |  |  |  |  |  |  |  |  |  |  |  |  |  |
|  | Perceived Stress | | -0.180 | -0.507 | 0.147 | 0.280 | 0.032 | -0.344 | 0.408 | 0.867 | 0.214 | 0.008 | 0.420 | 0.042 | -0.098 | -0.468 | 0.272 | 0.604 |
|  | Cognitive Functioning | | -0.052 | -0.299 | 0.195 | 0.679 | -0.020 | -0.269 | 0.229 | 0.873 | -0.057 | -0.196 | 0.082 | 0.424 | -0.029 | -0.276 | 0.218 | 0.817 |
|  | Depressive Symptoms | | 0.022 | -0.254 | 0.298 | 0.874 | 0.413 | 0.094 | 0.732 | 0.011 | 0.305 | 0.146 | 0.464 | <.001 | 0.349 | 0.016 | 0.682 | 0.041 |
|  | PTSD Symptoms | | 0.033 | -0.216 | 0.282 | 0.793 | -0.394 | -0.657 | -0.131 | 0.003 | -0.162 | -0.315 | -0.009 | 0.038 | -0.341 | -0.611 | -0.071 | 0.014 |
| Interpersonal Trauma | | |  |  |  |  |  |  |  |  |  |  |  |  |  |  |  |  |
|  | Adverse Childhood Experiences | | 0.311 | 0.125 | 0.497 | 0.001 | 0.160 | -0.036 | 0.356 | 0.110 | 0.132 | 0.012 | 0.252 | 0.031 | 0.263 | 0.067 | 0.459 | 0.008 |
|  | Intimate Partner Violence | | 0.176 | -0.034 | 0.386 | 0.100 | 0.237 | -0.004 | 0.478 | 0.054 | 0.080 | -0.079 | 0.239 | 0.323 | 0.294 | 0.043 | 0.545 | 0.021 |
|  | Sexual Assault | | -0.066 | -0.313 | 0.181 | 0.602 | 0.014 | -0.221 | 0.249 | 0.909 | 0.050 | -0.124 | 0.224 | 0.575 | -0.027 | -0.280 | 0.226 | 0.835 |
| Opioid Prescription | | |  |  |  |  | 0.433 | 0.247 | 0.619 | <.001 |  |  |  |  |  |  |  |  |
| Pain Intensity | | |  |  |  |  |  |  |  |  |  |  |  |  | 0.243 | 0.071 | 0.415 | 0.006 |
|  | | |  |  |  |  |  |  |  |  |  |  |  |  |  |  |  |  |
| Indirect Effects | | |  |  |  |  |  |  |  |  |  |  |  |  |  |  |  |  |
|  | Perceived Stress | |  |  |  |  | -0.078 | -0.221 | 0.065 | 0.288 |  |  |  |  | 0.052 | -0.013 | 0.117 | 0.118 |
|  | Cognitive Functioning | |  |  |  |  | -0.023 | -0.131 | 0.085 | 0.678 |  |  |  |  | -0.014 | -0.047 | 0.019 | 0.426 |
|  | Depressive Symptoms | |  |  |  |  | 0.010 | -0.115 | 0.130 | 0.874 |  |  |  |  | 0.074 | 0.005 | 0.143 | 0.033 |
|  | PTSD Symptoms | |  |  |  |  | 0.014 | -0.094 | 0.122 | 0.793 |  |  |  |  | -0.039 | -0.088 | 0.010 | 0.114 |
